# Avian Influenza A Viruses among Occupationally Exposed Populations, China, 2014–2016

**DOI:** 10.3201/eid2512.190261

**Published:** 2019-12

**Authors:** Chuansong Quan, Qianli Wang, Jie Zhang, Min Zhao, Qigang Dai, Ting Huang, Zewu Zhang, Shenghua Mao, Yifei Nie, Jun Liu, Yun Xie, Baorong Zhang, Yuhai Bi, Weifeng Shi, Peipei Liu, Dayan Wang, Luzhao Feng, Hongjie Yu, William J. Liu, George F. Gao

**Affiliations:** National Institute for Disease Control and Prevention, Chinese Center for Disease Control and Prevention, Beijing, China (C. Quan, J. Zhang, P. Liu, D. Wang, W.J. Liu, G.F. Gao);; Shandong First Medical University & Shandong Academy of Medical Sciences, Jinan, China (C. Quan, W. Shi);; Fudan University School of Public Health, Shanghai, China (Q. Wang, H. Yu);; Institute of Microbiology, Chinese Academy of Sciences, Beijing (M. Zhao, Y. Bi, G.F. Gao);; Jiangsu Provincial Center for Disease Control and Prevention, Nanjing, China (Q. Dai);; Sichuan Provincial Center for Disease Prevention and Control, Chengdu, China (T. Huang);; Dongguan Municipal Center for Disease Control and Prevention, Dongguan, China (Z. Zhang);; Shanghai Municipal Center for Disease Control and Prevention, Shanghai (S. Mao);; Henan Provincial Center for Disease Control and Prevention, Zhengzhou, China (Y. Nie);; Zaozhuang Center for Disease Control and Prevention, Zaozhuang, China (J. Liu);; Jiangxi Provincial Center for Disease Control and Prevention, Nanchang, China (Y. Xie);; Aviation General Hospital, Beijing (B. Zhang);; Chinese Center for Disease Control and Prevention, Beijing (L. Feng, G.F. Gao)

**Keywords:** seroepidemiology, seroprevalence, avian influenza virus, poultry worker, viruses, China, influenza, influenza A, occupational health, respiratory infections, zoonoses

## Abstract

To determine the seroprevalence and seroconversion of avian influenza virus (AIV) antibodies in poultry workers, we conducted a seroepidemiologic study in 7 areas of China during December 2014–April 2016. We used viral isolation and reverse transcription PCR to detect AIVs in specimens from live poultry markets. We analyzed 2,124 serum samples obtained from 1,407 poultry workers by using hemagglutination inhibition and microneutralization assays. We noted seroprevalence of AIV antibodies for subtypes H9N2, H7N9, H6N1, H5N1-SC29, H5N6, H5N1-SH199, and H6N6. In serum from participants with longitudinal samples, we noted seroconversion, with >4-fold rise in titers, for H9N2, H7N9, H6N1, H5N1-SC29, H6N6, H5N6, and H5N1-SH199 subtypes. We found no evidence of H10N8 subtype. The distribution of AIV antibodies provided evidence of asymptomatic infection. We found that AIV antibody prevalence in live poultry markets correlated with increased risk for H7N9 and H9N2 infection among poultry workers.

Human infection with avian influenza viruses (AIVs) has been reported in China since the late 1990s. Since then, human infections with subtypes H5N1, H5N6, H6N1, H7N4, H7N9, H9N2, and H10N8 have been reported continuously and are a substantial threat to public health in the country (*1**–**5*). Birds at wholesale and retail live poultry markets are recognized incubators for novel influenza virus subtypes (*6**–**9*). Because of special occupational characteristics, poultry workers are at a high risk for repeated exposure to AIV-infected poultry. Most case-patients with H7N9 infection have had a history of contact with live poultry, and poultry workers represent a substantial proportion of cases (*10*). Several studies on AIV seroprevalence in occupationally exposed populations suggest that asymptomatic or clinically mild AIV infections are extensively prevalent among poultry workers (*11**–**14*). A serologic study of AIV distribution among poultry workers could directly evaluate the potential for AIVs to cross the species barrier to infect humans and might illuminate the current understanding of AIV prevalence in live poultry markets (*15*).

Low pathogenicity avian influenza A(H9N2) virus is distributed widely in domestic poultry around the world. A systematic review reports H9N2 virus seroprevalence in avian-exposed populations ranges from 1% to 43% by hemagglutination inhibition (HI) assays (*16*). Since a 2013 H7N9 infection outbreak in China, caused by a novel reassortant influenza A(H7N9) virus and associated with severe human infections, seroprevalence of the H7N9 subtype has been reported to range from 6% to 14.9% in southern China (*17*,*18*). In a previous study, the seroprevalence of H5 subtype AIVs in poultry workers was relatively low, whereas a cross-sectional study conducted in Zhejiang Province reported a seroprevalence of 4.7% for H5N1 virus antibodies (*19*).

Few large-scale longitudinal seroepidemiologic studies have included multiple AIV subtypes in diverse epidemic regions, especially after emergence of novel subtypes. We conducted a prospective seroepidemiologic study in 7 representative areas across China to address gaps in the research. We characterized the seroprevalence profiles of 7 dominant human-infecting AIV subtypes among occupationally exposed workers in live poultry markets. Our aim was to further analyze human AIV infection risks for serotypes common in occupational exposure, including H5N1, H5N6, H6N1, H6N6, H7N9, H9N2, and H10N8 virus subtypes.

## Methods

### Ethics Approval

This study was approved by the Ethics Review Committee of the National Institute for Viral Disease Control and Prevention, Chinese Center for Disease Control and Prevention. The study was conducted in accordance with the principles of the Declaration of Helsinki and the standards of Good Clinical Practice as defined by the International Conference on Harmonization (https://www.ich.org).

### Study Design and Participants

**Figure 1:**
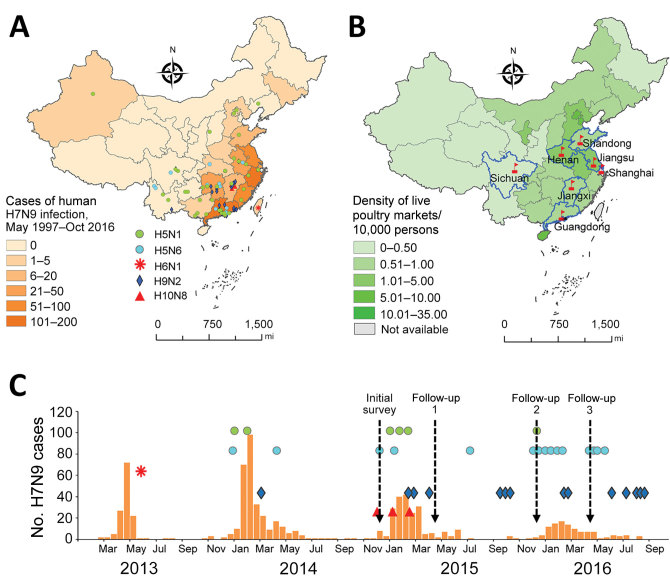
Temporal and spatial distribution of human infections with avian influenza A virus subtypes before and during serosurveillance, China. A) Geographic distribution of avian influenza A(H7N9) virus infection among humans in China during May 1997–October 2016. The number of case-patients in each province is based on data published by the World Health Organization and the National Health (https://www.who.int/influenza/human_animal_interface/avian_influenza/archive/en/) and Family Planning Commission of the Republic of China (http://www.nhc.gov.cn/jkj/s2907/new_list.shtml?tdsourcetag=s_pcqq_aiomsg). Density of shading represents the number of reported avian influenza H7N9 cases in humans in each province. Cases of other AIV subtype infections are represented by other symbols. B) Density of live poultry markets per 10,000 persons in each province included in the study, from data collected during 2013–2014. Red flags indicate locations of poultry markets selected for the serosurveillance study. C) Distribution of biweekly cases of human H7N9 infection before and during serosurveillance study. Orange bars indicate the number of biweekly cases of human H7N9 infection. Dashed lines indicate initial survey and follow-up dates for serosurveys, which were conducted before and after the third and fourth wave H7N9 epidemics. Reported cases of H5N1, H5N6, H6N1, H9N2, and H10N8 infection are noted with symbols as in panel A. AIV, avian influenza virus.

During December 2014–April 2016, we conducted a longitudinal seroepidemiologic study to assess asymptomatic AIV infection levels among poultry workers in China. We defined poultry workers as persons who repeatedly are exposed to poultry and work in wholesale or retail live poultry markets or in backyard farms, including wholesale sellers, retail sellers, transporters, processors, or feeders. The study included 1 municipality, Shanghai, and 6 provinces, Guangdong, Henan, Jiangsu, Jiangxi, Shandong, and Sichuan (Figure 1, panels A and B; Appendix). The study design included 4 serologic surveys. We collected whole blood samples from participating poultry workers at an initial visit in December 2014 and again during 3 consecutive follow-up visits in April 2015, December 2015, and April 2016 (Figure 1, panel C).

We used a standardized questionnaire to collect information at initial participant enrollment and updated participant information at subsequent visits. Participant information collected was demographic data, exposure variables, whether the worker experienced influenza-like illness within the previous month, and whether they received a seasonal influenza vaccination within the previous 12 months (Appendix).

**Figure 2:**
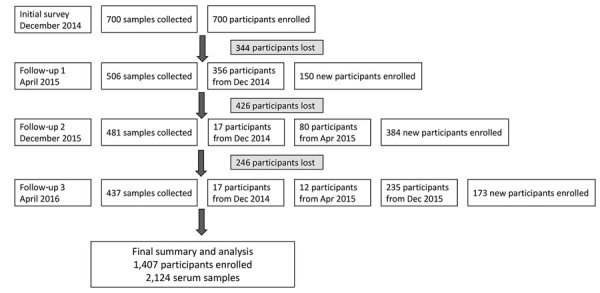
Flowchart of initial participant enrollment and follow-up distribution in 7 areas of China in a study of avian influenza virus seroprevalence during December 2014–April 2016.

Some poultry workers in China are short-term employees with high population mobility. We attempted to conduct follow-up studies with these employees through assistance from the market managers. To ensure the sample size, we enrolled new participants at each visit to the poultry markets (Figure 2).

We also recruited a control group of 216 outpatients with noninfectious diseases on physical examination at a general hospital in Beijing in October 2015. We collected 216 serum samples from the control group.

### Collection of Human Samples

We collected a single venous whole blood sample from each study participant at each visit by using a Vacutainer blood collection tube (Becton Dickinson, https://www.bd.com). We divided serum into 3 aliquots and froze at −80°C until testing.

### Serologic Assays

We tested participant serum samples for antibodies against H5N1, H5N6, H6N1, H6N6, H7N9, H9N2, and H10N8 virus subtypes, as well as for antibodies against seasonal influenza A(H1N1)pdm09 (pH1N1) and H3N2 viruses, to exclude cross-reactivity. We selected available representative antigens on the basis of their antigenic characteristics (Table 1) and analyzed the relevant phylogenetic relationship of hemagglutinin (HA) genes (Appendix Figures 1–5).

**Table 1:** Avian influenza A antigens used in serologic hemagglutinin inhibition and microneutralization assays, China*

Subtype	Virus strain	GISAID number
Avian influenza		
H5N1 clade 2.3.2.1c	A/chicken/Shanghai/02.12 HZ199-P/2015 (SH199)	EPI1544294
H5N1 clade 2.3.4.4	A/pigeon/Sichuan/NCXN29/2014 (SC29)	EPI590898
H5N6 clade 2.3.4.4	A/duck/Guangdong/04.22 DGCP069-O/2015	EPI660071
H6N1	A/Taiwan/2/2013	EPI459855
H6N6	A/duck/Guangxi/04.10 JX031/2015	EPI661887
H7N9	A/chicken/Guangdong/04.22 DGCP098-O/2015	EPI666285
H9N2	A/chicken/Guangdong/04.15 SZBAXQ005/2015	EPI661935
H10N8	A/chicken/Jiangxi /B18/2014	EPI1544302
Seasonal influenza		
H1N1(pdm09)	A/California/04/2009	EPI176470
H3N2	A/Beijing/CAS0001/2007	EPI1544286

*GISAID, https://www.gisaid.org.

We performed all serologic assays in a Biosafety Level 2 or 3 laboratory. First, we screened samples by using an HI assay for antibodies, as described previously (*20*). We tested serum samples at a starting dilution of 1:10, followed by a 2-fold dilution to the endpoint (Appendix). To confirm HI assay results, we performed a microneutralization (MN) assay on serum samples with an HI titer >1:20 to H5N1, H5N6, H6N1, H6N6, H7N9, or H10N8 subtypes and those with an HI titer >1:40 to H9N2, pH1N1, or H3N2 subtypes, as previously described (*20*). 

We used HI and MN cutoff values in accordance with previously published data (Appendix Table 1). We considered >1:20 as the cutoff value for HI and MN titers for positive tests for H5N1, H5N6, H6N1, H6N6, H7N9, and H10N8 virus subtypes (*11*,*12*,*21*) and considered >1:40 as the cutoff value for HI titer and >1:80 as the cutoff value for MN titer for positive tests for H9N2, pH1N1, and H3N2 virus subtypes (*22*,*23*). We set a stricter dilution cutoff value for the H9N2 virus subtype. An HI titer of 1:40 commonly is used and generally is an accepted value for influenza serologic assays used in detection of seasonal influenza and avian influenza H9 infection (*24*). We considered participants to have seroconversion when they had a >4-fold rise in antibody titer measured by HI assay between collection of >1 serum samples, plus an MN titer value of the later specimen being >1:20 or >1:80 for H9N2 subtype only.

### Isolation of AIVs from Environmental and Poultry Samples

For environmental and poultry samples, we used previously described sampling and detection methods (*25*). In brief, we randomly selected environmental sites and poultry to sample by using a multistage sampling strategy. We collected environmental samples by swabbing water troughs, floors, and drains in poultry enclosures and collected oropharyngeal and cloacal swabs from apparently healthy poultry. We isolated avian influenza viruses in 9- to 10-day-old specific pathogen–free chicken embryos by using viral isolation procedures and following World Health Organization guidelines (*20*). We further analyzed hemagglutinin-positive samples by using reverse transcription PCR (RT-PCR) to identify hemagglutinin (HA) and neuraminidase (NA) genetic subtypes (*20*). Except for Shandong Province, we detected AIVs from domestic poultry and live poultry market environments in all study areas.

### Data Analysis

Our analyses were based on seroepidemiologic studies for influenza published by Horby et al. (*26*). We assigned each participant a unique identifier and used all data collected with the questionnaire to establish a database. We performed a multivariate logistic regression model to evaluate independent risk factors associated with seroprevalence of antibodies in poultry workers. Risk factors evaluated were age; sex; occupational exposure factors, including processing, selling, transporting, and feeding poultry; and seropositivity to human influenza pH1N1 or H3N2 viruses. For logistic regression analysis, we estimated the maximum likelihood for the odds ratio (OR) and calculated 95% CIs by using the Wald χ^2^ test. We used binomial distribution to calculate 95% CIs of rate. We used Spearman correlation analysis to estimate the association between seroprevalence and local epidemic intensity of AIVs in live poultry markets by region. We used 2-tailed p values for all calculations and considered values <0.05 statistically significant. We performed statistical analyses by using SAS 9.4 (SAS Institute, Inc., https://www.sas.com).

## Results

### Participant Characteristics

We collected 2,124 serum samples from 1,407 participants from 1 municipality, Shanghai, and 6 provinces, Guangdong, Henan, Jiangsu, Jiangxi, Shandong, and Sichuan, in China. We had paired or serial serum samples from 652 participants who had >2 visits during the study period. The median age of participants with completed questionnaire information was 46 years (interquartile range [IQR] 36–52 years); 54.0% (1,147/2,124) of samples were from men. The most common category of poultry exposure was poultry seller. We did not see statistically significant differences in the distribution of demographic characteristics of participants, including sex and age, over the 4-period survey. In addition, 2.8% (59/2,124) of samples came from poultry workers who reported receiving a seasonal influenza vaccine within the previous 12 months (Table 2). 

**Table 2:** Characteristics of study participants in serosurveys for avian influenza viruses, China, 2014–2016*

Variables	2014 Dec, n = 700	2015 Apr, n = 506	2015 Dec, n = 481	2016 Apr, n = 437	Total, n = 2,124	χ^2^†	p value
Sex, no. (%)							
M	369 (52.7)	264 (52.2)	278 (51.8)	236 (54.0)	1,147 (54.0)	3.94	0.27
F	331 (47.3)	242 (47.8)	203 (42.2)	201 (46.0)	977 (46.0)		
Age, y, no. (%)‡							
<21	10 (1.4)	6 (1.2)	4 (0.8)	11 (2.5)	31 (1.5)	1.43	0.23
21–40	212 (30.3)	144 (28.5)	164 (34.1)	144 (33)	664 (31.3)		
41–60	394 (56.3)	308 (60.9)	254 (52.8)	232 (53.1)	1,188 (55.9)		
>60	78 (11.1)	47 (9.3)	55 (11.4)	50 (11.4)	230 (10.8)		
Missing data	6 (0.9)	1 (0.2)	4 (0.8)	0	11 (0.5)		
Median age (range)§	46 (38–52)	47 (38–52)	45 (35–52)	45 (35–52)	46 (36–52)	6.62	0.08
Type of poultry exposure, no. (%)¶							
Processing	155 (22.1)	107 (21.1)	118 (24.5)	94 (21.5)	474 (22.3)	27.88	0.006
Selling	423 (60.4)	332 (65.6)	299 (62.2)	191 (43.7)	1,243 (58.5)		
Transportation	39 (5.6)	31 (6.1)	24 (5)	21 (4.8)	115 (5.4)		
Feeding	191 (27.3)	125 (24.7)	124 (25.8)	93 (21.3)	533 (25.1)		
Others	59 (8.4)	35 (6.9)	25 (5.2)	48 (11)	167 (7.9)		
Missing data	0	0	2 (0.4)	0	2 (0.1)		
Length of poultry exposure, y (range)§	8 (3–15)	8 (3–15)	5 (2–10)	5 (3–10)	6 (3–13)	61.63	<0.001
Vaccinated against seasonal influenza, no. (%)	23 (3.3)	8 (1.6)	20 (4.2)	8 (1.8)	59 (2.8)	8.20	0.04

*Some participants participated in >1 survey. †By χ^2^ test, unless otherwise indicated. ‡By χ^2^_CMH_ test. Missing data were not calculated. §By Kruskal-Wallis test. ¶Most participants had multiple exposure types. Sums of percentages exceed 2,124. Missing data were not calculated.

Of the 216 participants in control group, the median age was 48 years (IQR 34–59 years); 45.8% were male. We saw no significant differences in their data compared with poultry workers (data not shown).

### Seroprevalence of Antibodies against AIVs

In the 2,124 samples, the overall seroprevalence of antibodies was 11.2% for H9N2 subtype and 3.9% for H7N9 subtype. Seroprevalence for H5Nx and H6Nx subtypes was lower, ranging from 1.3% to 2.1% for H5Nx and from 0.4% to 2.5% for H6Nx. We did not observe evidence of H10N8 infection (Table 3).

**Table 3:** Seroprevalence among poultry workers surveyed for avian influenza viruses, China, 2014–2016*

Antigen	No. (%, 95% CI) seropositive participants				
	2014 Dec, n = 700	2015 Apr, n = 506	2015 Dec, n = 481	2016 Apr, n = 437	Total, n = 2124
Avian influenza serotype					
H5N1-SH199	6 (0.9, 0.2–1.5)	6 (1.2, 0.2–2.1)	10 (2.1, 0.8–3.4)	6 (1.4, 0.3–2.5)	28 (1.3, 0.8–1.8)
H5N1-SC29	22 (3.1, 1.8–4.4)	17 (3.4, 1.8–4.9)	2 (0.4, 0.1–1.5)	3 (0.7, 0.1–2.0)	44 (2.1, 1.5–2.7)
H5N6	28 (4, 2.5–5.5)	11 (2.2, 0.9–3.4)	2 (0.4, 0.1–1.5)	1 (0.2, 0–1.3)	42 (2.0, 1.4–2.6)
H6N1	22 (3.1, 1.8–4.4)	21 (4.1, 2.4–5.9)	5 (1, 0.1–1.9)	5 (1.1, 0.4–2.6)	53 (2.5, 1.8–3.2)
H6N6	0 (0, 0–0.5)	0 (0, 0–0.7)	7 (1.5, 0.4–2.5)	1 (0.2, 0–1.3)	8 (0.4, 0.1–0.6)
H7N9	33 (4.7, 3.1–6.3)	36 (7.1, 4.9–9.4)	6 (1.3, 0.3–2.2)	7 (1.6, 0.4–2.8)	82 (3.9, 3.0–4.7)
H9N2	48 (6.9, 5.0–8.7)	59 (11.7, 8.9–14.5)	64 (13.3, 10.3–16.3)	66 (15.1, 11.7–18.5)	237 (11.2, 9.8–12.5)
Seasonal influenza serotype					
H1N1(pdm09)	94 (13.4, 10.9–16.0)	85 (16.8, 13.5–20.1)	90 (18.7, 15.2–22.2)	79 (18.1, 14.5–21.7)	348 (16.4, 14.8–18.0)
H3N2	237 (33.9, 30.4–37.4)	165 (32.6, 28.5–36.7)	199 (41.4, 37.0–45.8)	171 (39.1, 34.6–43.7)	772 (36.3, 34.3–38.4)

**Figure 3:**
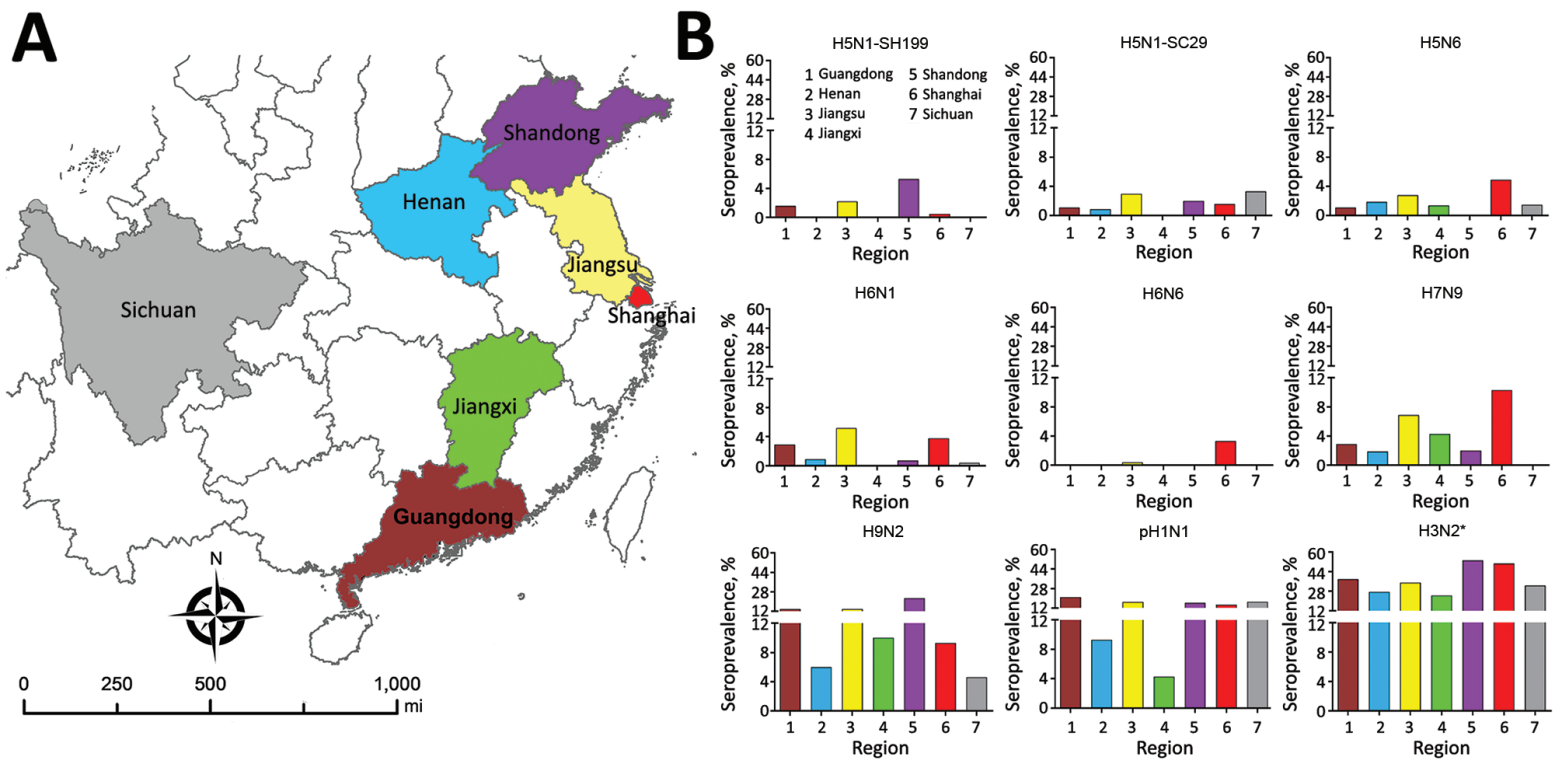
Avian influenza virus seroprevalence in the studied regions of China during December 2014–April 2016. A) Geographic areas included for serosurveillance: 1 municipality, Shanghai, and 6 provinces, Guangdong, Henan, Jiangsu, Jiangxi, Shandong, and Sichuan. B) Seroprevalence against avian influenza A virus subtypes in 4 cross-sectional surveys. Colors on map correspond to colors in bar graphs. *Seasonal influenza virus subtype.

The seroprevalence profile was geographically distinct (Figure 3). For example, in Shandong Province, H9N2 virus antibody seroprevalence was 23%, which was higher than in other provinces, especially Sichuan Province, which had only a 4.2% seroprevalence for this subtype. Provinces in the Yangtze River Delta, which were the first to report H7N9 infections in patients during the 2013 outbreak, exhibited higher seroprevalence rates compared with the other provinces. Shanghai had a rate of 10.3% and Jiangsu Province had a rate of 6.9%. In Sichuan Province, where a non–laboratory-confirmed H7N9-infected patient was reported before 2017, no participant tested positive for the H7N9 subtype. 

Seroprevalence of H5 and H6 subtypes among poultry workers also were different by region. Detected H5 subtypes included H5N1-SH199 clade 2.3.2.1c in 5.3% of samples from Shandong Province; H5N1-SC29 clade 2.3.4.4 in 3.0% of samples from Jiangsu Province and in 3.3% of samples from Sichuan Province; and H5N6 in 4.9% of samples from Shanghai. We detected H6N1 in 5.2% of samples from Jiangsu Province and in 3.8% from Shanghai and H6N6 in 3.3% of samples from Shanghai (Figure 3).

Among the 216 participants in the control group, we found no evidence of antibodies against H7N9 virus and a lower prevalence (3.7%) of antibodies against H9N2 virus than in the poultry workers. We observed no statistically significant differences in the prevalence of antibodies against other AIV subtypes between the control group and poultry workers (Appendix Table 2).

### Seroconversion of Antibodies against AIVs among Poultry Workers

**Figure 4:**
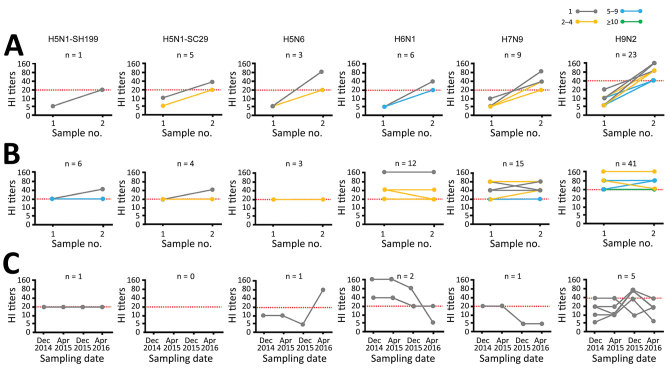
Seroconversion and persistent positivity for avian influenza virus (AIV) A subtypes based on HI titers in a cohort study in China during December 2014–April 2016. Each dot and line connection represents 1 participant. Red dashed lines represent positive cutoff for the HI titers; HI–positive samples were confirmed by a microneutralization assay. A) Comparison of paired samples from participants during 2 surveillance periods showing seroconversion for 6 AIV subtypes. Weighted lines and dots represent participants with seroconversion. B) Number of participants with >2 positive sample who were persistently seropositive for 6 AIV subtypes. Weighted lines and dots represent number of participants with seropositivity. C) Antibody titers of representative participants with >1 positive sample in the 4 serosurveys. HI, hemagglutinin inhibition.

We observed seroconversion in all AIV antigens during the study period, except the H10 subtype, which might represent a new asymptomatic AIV infection among poultry workers (Figure 4, panel A). Among 652 poultry workers with paired or serial serum samples during the study, 3.5% demonstrated seroconversion for H9N2 virus, 1.4% demonstrated seroconversion for H7N9 virus, and <1% demonstrated seroconversion for H5 or H6 viruses (Figure 4, panels B and C; Appendix Tables 3–9). Because we saw no evidence of H10N8 virus, we also saw no seroconversion for the subtype (Table 4; Figure 4, panel A).

**Table 4:** Seroconversion and persistently positive findings for avian influenza virus among 652 participants with paired or serial serum samples, China, 2014–2016*

Subtype	No. (%, 95% CI) participants	
	Seroconversion	Persistently positive
H5N1-SH199	1 (0.2, 0–0.9)	6 (0.9, 0.3–2.0)
H5N1-SC29	5 (0.8, 0.2–1.8)	4 (0.6, 0.2–1.6)
H5N6	3 (0.5, 0.0–1.3)	3 (0.5, 0.0–1.3)
H6N1	6 (0.9, 0.3–2.0)	12 (1.8, 0.8–2.9)
H6N6	4 (0.6, 0.2–1.7)	0 (0.0, 0.0–0.6)
H7N9	9 (1.4, 0.5–2.3)	15 (2.3, 1.1–3.5)
H9N2	23 (3.5, 2.1–4.9)	41 (6.3, 4.4–8.2)
H10N8	0 (0–0.6)	0 (0–0.6)

Some participants showed consistently seropositive results, 15 for H7N9 subtype and 41 for H9N2 subtype and a few each for H5N1, H5N6, and H6N1 subtypes (Figure 4, panel B). One participant (no. 14.12GD72) showed HI titers at 1:20 and MN titers at 1:160 to H5N1-SH199 subtype in 4 consecutive surveys (Figure 4, panel C).

### Risk Analysis for Asymptomatic AIV Infections

In the multivariable analysis, we identified demographic and occupational risk factors for poultry workers with asymptomatic infections. For instance, the demographic classification female (adjusted OR [aOR]  2.2, 95% CI 1.4–3.6), and occupational classification poultry seller (aOR 4.1, 95% CI 2.2–7.7) appear to be risk factors for H7N9 infection. For H9N2 subtype, female (aOR 1.6, 95% CI 1.2–2.1) and poultry seller (aOR 1.9, 95% CI 1.4–2.6) appear to be risk factors for infection. In addition, the number of years working in poultry-related occupations was associated with seroprevalence. In particular, samples from workers reporting >3 years of exposure were associated with seroprevalence of H9N2 subtype. Factors associated with increased risk for H5 infections included being >55 years of age, being exposed to ducks, or being exposed to ill or dead poultry (Table 5).

**Table 5:** Risk analysis for seropositive participants in surveys for avian influenza subtypes among poultry workers, China, 2014–2016*

Subtypes and variables	Seropositive, no. (%)	Seronegative, no. (%)	p value†	OR (95% CI)	Adjusted OR (95% CI)	
H5Nx‡						
Age, y						
<35	8 (10.8)	442 (21.7)	<0.001	Referent	Referent	
35–55	40 (54.1)	1,231 (60.4)		1.8 (0.8–3.9)	2.3 (1.0–4.9)	
>55	26 (35.1)	366 (18.0)		3.9 (1.8–8.8)	4.7 (2.1–10.7)	
Exposed to ducks						
Yes	34 (45.3)	651 (31.8)	0.014	1.8 (1.1–2.8)	1.6 (1.0–2.5)	
No	41 (54.7)	1,398 (68.2)		Referent	Referent	
Exposed to ill or dead poultry						
Yes	15 (20.0)	221 (10.8)	0.013	2.1 (1.2–3.7)	2.3 (1.3–4.2)	
No	60 (80.0)	1826 (89.2)		Referent	Referent	
Seropositivity for H1N1(pdm09) virus						
Positive	24 (32.0)	316 (16.4)	<0.001	2.6 (1.6–4.3)	3.1 (1.8–4.5)	
Negative	51 (68.0)	1,733 (84.6)		Referent	Referent	
H7N9						
Sex						
F	53 (64.6)	924 (45.2)	<0.001	2.2 (1.4–3.5)	2.2 (1.4–3.6)	
M	29 (35.4)	1,118 (54.8)		Referent	Referent	
Poultry seller§						
Yes	70 (85.4)	1,173 (57.5)	<0.001	4.3 (2.3–8.0)	4.1 (2.2–7.7)	
No	12 (14.6)	867 (42.5)		Referent	Referent	
No. years of work at live poultry market						
<3	11 (13.4)	561 (27.5)	0.017	Referent	Referent	
3–10	46 (56.1)	924 (45.3)		2.0 (1.1–3.5)	1.8 (1.0–3.2)	
>10	25 (30.5)	557 (27.3)		1.7 (0.9–3.2)	1.3 (0.7–2.5)	
Seropositivity for seasonal H3N2 virus						
Positive	44 (53.7)	743 (36.4)	0.002	2.0 (1.3–3.2)	1.9 (1.2–2.9)	
Negative	38 (46.4)	1,299 (63.6)		Referent	Referent	
H9N2						
Age, y§						
<35	56 (23.6)	394 (21.0)	0.004	2.1 (1.3–3.4)	1.9 (1.1–3.3)	
35–55	156 (65.8)	1,115 (59.4)		2.1 (1.3–3.2)	1.6 (1.0–2.5)	
>55	25 (10.6)	367 (19.6)		Referent	Referent	
Sex						
F	134 (56.5)	843 (44.7)	<0.001	1.6 (1.2–2.1)	1.6 (1.2–2.1)	
M	103 (43.5)	1,044 (55.3)		Referent	Referent	
Poultry seller§						
Yes	175 (73.8)	1,068 (56.7)	<0.001	2.2 (1.6–2.9)	1.9 (1.4–2.6)	
No	62 (26.2)	817 (43.3)		Referent	Referent	
Poultry processor§						
Yes	67 (28.3)	407 (21.6)	0.02	1.4 (1.1–1.9)	1.3 (1.0–1.7)	
No	170 (71.7)	1,478 (78.4)		Referent	Referent	
No. years of work at live poultry market						
<3	37 (15.6)	535 (28.4)	<0.001	Referent	Referent	
3–10	126 (53.2)	844 (44.7)		2.6 (1.8–3.7)	2.4 (1.6–3.5)	
>10	74 (31.2)	508 (26.9)		2.2 (1.5–3.2)	3.0 (1.3–3.1)	
H6N1						
Seropositivity for H1N1(pdm09) virus						
Positive	19 (35.9)	321 (15.5)	<0.001	3.0 (1.7–5.4)	3.0 (1.7–5.4)	
Negative	34 (64.1)	1,750 (84.5)		Referent	Referent	
Seropositivity for H9N2 virus						
Positive	13 (24.5)	224 (10.8)	0.002	2.7 (1.4–5.1)	2.6 (1.4–5.0)	
Negative	40 (75.5)	1,847 (89.2)		Referent	Referent	

*Results represent only statistically significant factors from analysis of questionnaire data. †By χ^2^ test. ‡Combined the H5N1-SC29 and H5N6 data. §Missing data.

Our study revealed a correlation between the presence of antibodies and seasonal influenza virus infection. We saw an association between the presence of pH1N1 virus antibodies and increased seropositivity for H5N1 or H5N6 subtypes, and between occurrence of seasonal H3N2 virus antibodies in humans and positive antibody titers for H7N9 virus subtype. We also saw a positive association between elevated H6N1 seropositivity and the presence of antibodies against pH1N1 (aOR 3.0, 95% CI 1.7–5.4) and H9N2 (aOR 2.6, 95% CI 1.4–5.0) subtypes (Table 5). Seasonal influenza vaccination history was not a significant risk factor for elevated AIV antibody titers, perhaps because of low vaccination rates.

### AIV Circulation in Poultry and Markets

We collected 6,207 samples from poultry and the environment for AIV screening and detection in this study. In Shanghai, 4.1% (20/493) of samples were positive for H7N9 subtype, as were 8.6% (41/476) of samples from Jiangsu Province. However, only 0.6% (15/2,308) of samples from Jiangxi Province, 0.6% (12/2,158) of samples from Guangdong Province, and 0.2% (1/516) of samples from Sichuan Province were positive for H7N9 subtype (Appendix Table 10).

For H9N2 subtype, 14.4% (71/493) of samples from Shanghai, 9.5% (45/476) from Jiangsu Province, and 8.3% (180/2,158) of samples from Guangdong Province were positive. However, only 4.4% (102/2,308) of samples from Jiangxi Province and 5.5% (14/256) from Henan Province were positive for H9N2 (Appendix Table 10).

Exploring the correlation between AIV circulation in poultry and seroprevalence in workers in live poultry markets revealed a correlation coefficient of 0.8 (p = 0.04) for H7N9 virus and 0.5 (p = 0.28) for H9N2 virus, indicating that prevalence of local AIVs was statistically correlated with H7N9 subtype seroprevalence. Our results also revealed that AIV prevalence in the different provinces was a key determinant of seroprevalence in the corresponding poultry workers. However, we did not observe a similar trend with other seroepidemic subtypes.

## Discussion

We conducted a longitudinal seroepidemiologic study of occupationally exposed poultry workers in China during December 2014–April 2016. We investigated antibody profiles of 7 AIV subtypes that have crossed the species barrier to infect humans, H5N1, H5N6, H6N1, H7N9, H9N2 and H10N8 subtypes, and H6N6 subtype, which is a potential risk to humans. We assessed seroconversion by analyzing paired serum samples from poultry workers and detecting AIV in poultry and the environment in live poultry markets.

H9N2 virus, which plays a role at the animal–human interface, serves as gene donor for H7N9 and H10N8 viruses that infect humans (*27*). We used a Y280/G9 lineage antigen isolated in samples from Guangdong Province in 2015 as a reference, and its seroprevalence was higher than all other AIV subtypes in our study (Appendix Figure 4). Previous serologic studies also have reported that this strain’s seroprevalence consistently is higher than other AIV subtypes in most provinces surveyed in China, reflecting the association between prevalent asymptomatic infections and frequent poultry exposure (*12*,*16*,*28*). 

Overall, seroprevalence of antibodies against H9N2 subtype in this study was higher than reported in previous serologic studies in China and the seroprevalence was highest in Shandong Province compared with other provinces. Li et al. reported a 3.04% seroprevalence between 2009 and 2011 in occupationally exposed populations (*29*), and Yu et al. reported 4.6% of poultry workers in their study had antibodies against H9N2 virus in 2013 (*30*). Another previous serologic study in Tai’an, a Shandong Province, reported the prevalence of antibodies against H9 subtypes among poultry workers was <8.5% during January 2011–December 2013 (*31*). Because no uniform standard antibody titer cutoff is available for H9N2 seropositivity, we used a stricter cutoff value for HI titers, >1:40, and for MN titers, >1:80, for seroprevalence to avoid overestimation and reduce cross-reactivity with seasonal influenza viruses (*32*).

The higher seroprevalence in Shandong Province could be explained by 2 possibilities. Participants in this province were all poultry sellers in live poultry markets, an occupation that we noted as a statistically high risk factor for seroprevalence. Shandong is ranked as the one of the largest egg-producing provinces in China, and it has a high prevalence of H9N2 in local chicken flocks (*33**,**34*), which could indicate that more people are exposed to AIV from the poultry industry in general. 

Logistic regression analysis of risk factors showed that occupational characteristics might increase risk for infection. Seropositive participant characteristics and related AIV information provided pivotal seroevidence for subclinical AIV infection risk factors. We noted that the participant characteristics female and poultry seller were risk factors for H7N9 and H9N2 infection, which coincides with results of previous studies (*18*,*35*). Further risk factor analysis indicated that seropositivity for pH1N1 virus was a risk factor for H5 infections with H5N1 and H5N6 subtypes and for H6N1 infection and that seropositivity for H3N2 subtype was a risk factor for H7N9 infection. In addition, seroprevalence for H6N1 infection also was affected by seropositivity for H9N2 subtypes. Our results might be explained partially by cross-reactivity between HA antigen from different AIV subtypes (*36*,*37*). We noted that the prevalence of H7N9 and H9N2 viruses in poultry from local markets was associated closely with seroprevalence for these subtypes in poultry workers. We also noted that the low seasonal influenza vaccination rate (2.8%) in poultry workers might have a limited effect on potential cross-reactions between pH1N1 and H5 subtypes and between H3N2 and H7N9 subtypes.

We observed higher prevalence for certain AIV subtypes and seroprevalence for certain AIV antibodies in live poultry markets, providing further evidence of cross-species transmission from birds to humans. Since the H7N9 outbreaks of 2013, consensus that AIV was transmitted from birds to humans led the government of China to implement epidemic control measures. The measures, such as closing live poultry markets during influenza season, cleaning and disinfecting live poultry markets daily, and vaccinating poultry, have effectively reduced the chances for human exposure to AIV-contaminated environments and ill poultry (*38*,*39*). Our results demonstrate that epidemic control measures aimed at live poultry markets, including their closure, can be highly effective in human AIV infection control (*9*,*38*).

Many participants with seropositivity were residents of southern and eastern provinces. Several determinants could account for this observation. First, the high density of live poultry markets, high population density, and expansive live poultry transportation network in these regions could favor large-scale and transboundary AIV spread in poultry, thereby increasing the risk for human infection (*40*). Second, these regions are rich in water resources, including the Yangtze and Pearl Rivers, as well as many lakes, which are natural habitats for waterfowl and wild birds that serve as natural hosts for various AIV subtypes, including H5Nx and H9N2 viruses, and that continually generate biological threats to public health (*41*,*42*). Studies suggest that migratory birds play a role in the evolution and spread of various zoonotic agents, and southeast China is located along the East Asian-Australian flyway, a migratory route for many bird species (*43*,*44*).

Our study had several limitations. Despite serious efforts to collect samples from the same participants during follow-up sampling, movement of vendors and poultry workers from target poultry markets reduced the possibility of obtaining paired samples. In addition, the relatively small sample size and use of only 1 location for the control group, Beijing in 2015, could be potential sources of bias.

In conclusion, our study provides serologic evidence of subclinical human AIV infection in an occupationally exposed population of poultry workers and corresponding AIV infection risk factors. Because novel influenza viruses continue to emerge, our results show the need for enhanced etiologic surveillance of AIVs in live poultry markets and humans. Implementing poultry vaccination would also reduce human infection risk. Finally, our results demonstrate the need for active surveillance to foresee dynamic AIV epidemics and inform influenza vaccine development.

Appendix Additional information on avian influenza A viruses among occupationally exposed populations, China, 2014–2016.
