# Supplementary material for: Avian Influenza A Viruses among Occupationally Exposed Populations, China, 2014–2016
Source: Emerg Infect Dis. 2019 Dec;25(12):2215–25. doi: 10.3201/eid2512.190261 (PMC6874249; doi:10.3201/eid2512.190261)
Supplement: Appendix — Additional information on avian influenza A viruses among occupationally exposed populations, China, 2014–2016. [file 19-0261-Techapp-s1.pdf]

Article DOI: https://doi.org/10.3201/eid2512.190261

Avian Influenza A Viruses among Occupationally Exposed Populations, China, 2014–2016

Appendix

Supplementary Methods

Selection of Study Regions

Shanghai and the 6 provinces selected for study all had previously reported human infections with avian influenza virus (AIV) or outbreaks of AIVs in poultry; a high density of poultry and population; were located in different key regions of China, Jiangsu and Shanghai in the east, Sichuan in the west, Jiangxi and Guangdong in the south, and Henan and Shandong in the north; and had a local Chinese Center for Disease Control and Prevention willing to take part in this study.

Questionnaire

We developed a structured questionnaire and administered it to each participant during a face-to-face interview after obtaining their consent to participate in the study. Questionnaire information included demographic characteristics (name, sex, age, work address); telephone number; occupational exposure history variables, including type of exposure (processing, selling, transportation, feeding and others), duration of work exposure to poultry, and which species of poultry they were exposed to; status of seasonal influenza vaccination within last 1 year; whether participant had influenza-like illness within 1 month.

Analysis of Antigenic Characteristic

Based on the antigenic and genetic characteristics of avian influenza A(H5) reported by the World Health Organization (WHO) and previous studies (*1–3*), viruses circulating and characterized during September 2014–September 2016 in mainland China belonged to clades 2.3.2.1c and 2.3.4.4 of HA genes. The 2.3.2.1c clade contained 2 candidate virus strains A/chicken/Ghana/20/2015 and A/chicken/Guiyang/1153/2016. Both viruses are located in different groups and have different antigenic characteristics. We selected an A/chicken/Shanghai/02.12 HZ199-P/2015 (H5N1-SH199) strain based on the genetic similarity. Although A(H5) 2.3.4.4 clades are the more prevalent strains and A/Sichuan/26221/2014 is the recommended reference strain, this clade also could be divided into 2 groups. Based on the prevalence of viruses, we selected A/pigeon/Sichuan/NCXN29/2014 (H5N1-SC29) and A/duck/Guangdong/04.22DGCP069-O/2015 (H5N6) for testing antigens, which were slightly different (Appendix, Figures 1 and 2).

According to data published by WHO, the available A(H7N9) candidate influenza vaccine virus is A/Anhui/1/2013 or A/Shanghai/2/2013. Additionally, prevalent viruses of H7N9 in China have similar antigenicity, according to a report by Wang, et al. (*4*). Therefore, the antigens used in our study could represent the prevalent H7N9 virus (Appendix, Figure 3).

According to data published by WHO, the available influenza A(H9N2) candidate vaccine virus is the A/chicken/Hong Kong/G9/1997 or derivative virus A/Hong Kong/308/2014-like (*1*). Additionally, the G9 clade could be divided into subgroups depending on antigenicity as reported by Li, et al. (*5*). Therefore, the antigens we used could represent the prevalent H9N2 virus (Appendix, Figure 4). On the basis of previous studies (*6*,*7*), antigens of H6Nx were similar, so we believed viruses used in this study could cover prevalent virus subtypes (Appendix, Figure 5).

Molecular and Phylogenetic Analyses

Through the antigenic analysis of the different AIV subtypes based on the reports and phylogenetic methods described previously (*1*), we performed a detailed phylogenetic analysis for HA gene segments with other prevailing AIVs. We concluded that selected strains were representative (Appendix, Figures 1–5**)**.

Hemagglutination Inhibition (HI) Assay

We inactivated antigens by using 0.05% β-propiolactone for HI assay. We treated 100 μL serum samples with 300 μL of receptor-destroying enzyme (RDE) and incubated in a water bath at 37°C for 16–18h. Then, we inactivated RDE(II) (Denka Seiken Co. Ltd, https://denka-seiken.com) at 56°C for 30 minutes before adding 100 μL phosphate buffer saline. To remove nonspecific inhibitors, we added 25 μL of packed chicken red blood cells (RBCs) to 500 μL of the mixtures. We incubated solutions in a 37°C water bath for 1h.

We performed serial 2-fold dilutions of RDE-treated serum from 1:10–1:160 in 25 μL phosphate buffer saline a 96-well microtitration plate. We added 25 μL of 4 haemagglutinin unit antigens and incubated at room temperature for 45 min. Then we added 50 μL of the RBC solution and incubated again for 45 minutes at room temperature. We considered titers accurate when hemagglutination was completely inhibited. We used negative and positive serum control samples (rabbit antibodies against the specific virus antigen) for each assay. We took titers as the reciprocal of the dilution levels of the wells. We assigned final titers of <1:10 a value of 1:5.

Microneutralization (MN) Assay

We heat inactivated serum samples at 56°C for 30 minutes and then conducted serial 2-fold dilutions from 1:20–1:640 in triplicate. We diluted equal volumes of heat-inactivate serum and virus to the 50% tissue culture infective dose ($TCID_{50}$) at 200, added these together, and incubated at 37°C for 1 h. We used the Reed-Muench method to determine the $TCID_{50}$/100 μL. We transferred the mixture to a confluent layer of Madin-Darby canine kidney cells and

incubated for 60 h at 37°C with 5% CO_2. Then we tested virus hemagglutination activity in 0.5% RBCs and considered absence of hemagglutination as a positive result for antibodies to the antigen (*8*). We used rabbit antibodies raised against homologous viruses as positive controls for assays. In each assay, we used negative, positive, and cell control serum, and virus back titration. We used the same negative and positive controls used in the MN assay that we used in HI assays. We defined the microneutralization titer as the highest dilution of serum that completely inhibited absence of hemagglutination in 50% of the wells. We assigned final titers <1:20 a value of 1:10.

References

1. World Health Organization. Antigenic and genetic characteristics of zoonotic influenza viruses and candidate vaccine viruses developed for potential use in human vaccines. Geneva: The Organization; 2019 Feb 21. [cited 2019 Aug 13]. http://www.who.int/influenza/vaccines/virus/characteristics_virus_vaccines/en

2. Bi Y, Chen Q, Wang Q, Chen J, Jin T, Wong G, et al. Genesis, evolution and prevalence of H5N6 avian influenza viruses in China. Cell Host Microbe. 2016;20:810–21. PubMed https://doi.org/10.1016/j.chom.2016.10.022

3. Yang L, Zhu W, Li X, Bo H, Zhang Y, Zou S, et al. Genesis and dissemination of highly pathogenic H5N6 avian influenza viruses. J Virol. 2017;91:e02199-16. PubMed https://doi.org/10.1128/JVI.02199-16

4. Wang D, Yang L, Zhu W, Zhang Y, Zou S, Bo H, et al. Two outbreak sources of influenza A (H7N9) viruses have been established in China. J Virol. 2016;90:5561–73. PubMed https://doi.org/10.1128/JVI.03173-15

5. Li X, Shi J, Guo J, Deng G, Zhang Q, Wang J, et al. Genetics, receptor binding property, and transmissibility in mammals of naturally isolated H9N2 Avian Influenza viruses. PLoS Pathog. 2014;10:e1004508. PubMed https://doi.org/10.1371/journal.ppat.1004508

6. Xin L, Bai T, Zhou JF, Chen YK, Li XD, Zhu WF, et al. Seropositivity for avian influenza H6 virus among humans, China. Emerg Infect Dis. 2015;21:1267–9. PubMed https://doi.org/10.3201/eid2107.150135

7. Wang G, Deng G, Shi J, Luo W, Zhang G, Zhang Q, et al. H6 influenza viruses pose a potential threat to human health. J Virol. 2014;88:3953–64. PubMed https://doi.org/10.1128/JVI.03292-13

8. Liu WJ, Tan S, Zhao M, Quan C, Bi Y, Wu Y, et al. Cross-immunity against avian influenza A(H7N9) virus in the healthy population is affected by antigenicity-dependent substitutions. J Infect Dis. 2016;214:1937–46. PubMed https://doi.org/10.1093/infdis/jiw471

9. Wang M, Fu CX, Zheng BJ. Antibodies against H5 and H9 avian influenza among poultry workers in China. N Engl J Med. 2009;360:2583–4. PubMed https://doi.org/10.1056/NEJMc0900358

10. Bai T, Zhou J, Shu Y. Serologic study for influenza A (H7N9) among high-risk groups in China. N Engl J Med. 2013;368:2339–40. PubMed https://doi.org/10.1056/NEJMc1305865

11. Gomaa MR, Kayed AS, Elabd MA, Zeid DA, Zaki SA, El Rifay AS, et al. Avian influenza A(H5N1) and A(H9N2) seroprevalence and risk factors for infection among Egyptians: a prospective, controlled seroepidemiological study. J Infect Dis. 2015;211:1399–407. PubMed https://doi.org/10.1093/infdis/jiu529

12. Chen Y, Zheng Q, Yang K, Zeng F, Lau SY, Wu WL, et al. Serological survey of antibodies to influenza A viruses in a group of people without a history of influenza vaccination. Clin Microbiol Infect. 2011;17:1347–9. PubMed https://doi.org/10.1111/j.1469-0691.2011.03538.x

13. Qi W, Su S, Xiao C, Zhou P, Li H, Ke C, et al. Antibodies against H10N8 avian influenza virus among animal workers in Guangdong Province before November 30, 2013, when the first human H10N8 case was recognized. BMC Med. 2014;12:205. PubMed https://doi.org/10.1186/s12916-014-0205-3

Appendix Table 1. Avian influenza A virus subtypes, reference strains, cutoff values for hemagglutinin and microneutralization assays, and reference studies for an investigation of avian influenza viruses among poultry workers, China, 2014–2016*

Subtypes	Reference strains	HI cutoff	MN cutoff	Reference no., study location
A(H1N1)pdm09	A/California/04/2009	40	80	*8*, *China*
H3N2	A/Beijing/CAS0001/2007	40	80	*8*, China
H5N1	A/chicken/Shanghai/02.12 HZ199-P/2015 (SH199)	20	NA	*9*, China
H5N1	A/pigeon/Sichuan/NCXN29/2014 (SC29)	20	NA	*9*, China
H5N6	A/duck/Guangdong/04.22 DGCP069-O/2015	20	NA	*9*, China
H6N1	A/Taiwan/2/2013	20	20	*6*, China
H6N6	A/duck/Guangxi/04.10 JX031/2015	20	20	*6*, China
H7N9	A/chicken/Guangdong/04.22 DGCP098-O/2015	20	40	*10*, China
H9N2	A/chicken/Guangdong/04.15 SZBAXQ005/2015	40	80	*11*, Egypt;*12*, China
H10N8	A/chicken/Jiangxi/B18/2014	20	20	*13*, China

*HI, hemagglutinin assay; MN, microneutralization assay; NA, not applicable.

Appendix Table 2. Seroprevalence of avian influenza A virus subtypes in the control group and study population of occupationally-exposed poultry workers*

Subtypes	Participants	Seroprevalence, no.(%)	p value
H5N1-SH199	Control group	3 (1.4)	0.2
	Poultry workers	28 (1.3)	
H5N1-SC29/H5N6	Control group	3 (1.4)	0.04
	Poultry workers	75 (3.5)	
H6N1	Control group	2 (0.9)	0.07
	Poultry workers	53 (2.5)	
H6N6	Control group	0 (0)	0.5
	Poultry workers	8 (0.4)	
H7N9	Control group	0 (0)	<0.01
	Poultry workers	82 (3.9)	
H9N2	Control group	8 (3.7)	<0.001
	Poultry workers	237 (11.2)	
A(H1N1)pdm09	Control group	47 (23.6)	0.03
	Poultry workers	340 (16.0)	
H3N2	Control group	75 (34.7)	0.6
	Poultry workers	773 (36.4)	

*No. of general population is 216; No. of poultry workers is 2124.

Appendix Table 3. Participants with seroconversion and persistently positive titers for avian influenza H5N1-SH199, China, 2014–2016*

			Length of	Dec 2014		Apr 2015		Dec 2015		Apr 2016	
Subject ID	Age, y/sex	Occupation	exposure, y	HI	MN	HI	MN	HI	MN	HI	MN
Seroconversion											
15.8SH14	47, F	Seller	16	NA†	NA	**5**	**ND**	**20**	**40**	NA	NA
Persistently positive											
14.11JS124	57, M	Seller	30	20	20	20	20	NA	NA	NA	NA
14.11JS31	30, F	Processor, seller	3	20	40	40	40	NA	NA	NA	NA
14.12GD72	34, M	Seller	5	20	160	20	160	20	160	20	160
16.1SD031	33, F	Processor, seller	10	NA	NA	NA	NA	20	40	20	40
16.1SD042	58, M	Processor, seller	26	NA	NA	NA	NA	20	80	20	80
16.1JS24	65, F	Feeder	2	NA	NA	NA	NA	20	40	20	40

*Bold text represents titers exhibiting seroconversion. HI, hemagglutinin inhibition assay; ID, identification; MN, microneutralization assay; NA, not available; ND, not detected.

Appendix Table 4. Participants with seroconversion and persistently positive titers for avian influenza H5N1-SC29, China, 2014–2016*

Subject ID	Age, y/sex	Occupation	Length of exposure, y	Dec 2014		Apr 2015		Dec 2015		Apr 2016	
				HI	MN	HI	MN	HI	MN	HI	MN
Seroconversion											
14.11SC60	61/M	Seller	5	**5**	**ND**	**20**	**40**	NA†	NA	NA	NA
14.11SC64	62/M	Seller	2	**5**	**ND**	**20**	**40**	NA	NA	NA	NA
14.11SC55	52/F	Seller	6	**10**	**40**	**40**	**80**	NA	NA	NA	NA
14.11JSWX54	48/F	Seller	24	**5**	**ND**	**20**	**80**	NA	NA	NA	NA
16.1SD76	39/M	Processor, seller	17	NA	NA	NA	NA	**5**	**ND**	**20**	**40**
Persistently positive											
14.11JS73	45/F	Seller	10	20	20	20	40	NA	NA	NA	NA
14.11JSWX39	43/F	Others	20	20	80	40	40	NA	NA	NA	NA
14.11SC86	50/M	Seller, transporter	5	20	40	20	40	NA	NA	NA	NA
14.11JSWX40	49/M	Other	30	20	40	20	40	NA	NA	NA	NA

*Bold text represents titers exhibiting seroconversion. HI, hemagglutinin inhibition assay; ID, identification; MN, microneutralization assay; NA, not available; ND, not detected.

Appendix Table 5. Participants with seroconversion and persistently positive titers for avian influenza H5N6, China, 2014–2016*

Participant ID	Age,y/sex	Occupation	Length of exposure, y	Dec 2014		Apr 2015		Dec 2015		Apr 2016	
				HI	MN	HI	MN	HI	MN	HI	MN
Seroconversion											
14.11JS67	50, F	Seller	10	**5**	**ND**	**20**	**20**	NA	NA	NA	NA
14.11SC85	57, F	Seller, transporter	30	**5**	**ND**	**20**	**20**	NA	NA	NA	NA
14.12GD72	34, M	Processor, seller	5	10	ND	10	ND	**5**	**ND**	**80**	**40**
Persistently positive											
14.11SC98	61, M	Processor	14	20	40	20	40	NA	NA	NA	NA
14.11JS123	58, F	Processor	4	20	80	20	160	NA	NA	NA	NA
14.11JS122	43, F	Other	2	20	20	20	20	NA	NA	NA	NA

*Bold text represents titers exhibiting seroconversion. HI, hemagglutinin inhibition assay; ID, identification; MN, microneutralization assay; NA, not available; ND, not detected.

Appendix Table 6. Participants with seroconversion and persistently positive titers for avian influenza H6N1, China, 2014–2016*

Participant ID	Age, y/sex	Occupation	Length of exposure, y	Dec 2014		Apr 2015		Dec 2015		Apr 2016	
				HI	MN	HI	MN	HI	MN	HI	MN
Seroconversion											
14.11JS82	47/F	Feeder	10	**5**	**ND**	**20**	**40**	NA	NA	NA	NA
14.12HN91	40/M	Other	5	**5**	**ND**	**40**	**80**	NA	NA	NA	NA
14.12 GD7	61/M	Other	2	5	ND	**5**	**ND**	**20**	**160**	NA	NA
15.5JS1	59/F	Feeder	23	**5**	**ND**	**20**	**40**	NA	NA	NA	NA
16.1JS112	59/M	Seller	13	NA	NA	NA	NA	**5**	**ND**	**20**	**40**
16.1JS54	28/M	Feeder	3	NA	NA	NA	NA	**5**	**ND**	**20**	**80**
Persistently positive											
14.11JS39	43/F	Other	20	20	40	20	40	NA	NA	NA	NA
14.11JS6	40/F	Processor, seller	12	40	320	20	80	NA	NA	NA	NA
14.11JSWX06	27/M	Seller	5	40	80	40	80	NA	NA	NA	NA
14.11JS99	59/M	Feeder	2	40	40	40	40	NA	NA	NA	NA
14.11JS102	33/M	Feeder	3	40	80	40	40	NA	NA	NA	NA
14.11JS12	60/M	Processor, seller	13	40	40	40	40	NA	NA	NA	NA
14.11JS85	50/M	Feeder	6	20	80	20	80	NA	NA	NA	NA
14.11JSWX49	43/F	Selling	24	20	40	20	40	NA	NA	NA	NA
16.1JS24	65/F	Feeding	2	NA	NA	NA	NA	20	160	20	160
14.12 GD 09	54/F	Seller	4	40	20	NA	NA	10	ND	20	20
14.12GD72	34/M	Seller	5	160	40	160	40	80	20	5	ND
14.12GD115	28/F	Seller	3	40	40	40	40	20	80	20	80

*Bold text represents titers exhibiting seroconversion. HI, hemagglutinin inhibition assay; ID, identification; MN, microneutralization assay; NA, not available; ND, not detected.

Appendix Table 7. Participants with seroconversion for avian influenza H6N6, China, 2014–2016*

Participant ID	Age, y/sex	Occupation	Length of exposure, y	Dec 2014		Apr 2015		Dec 2015		Apr 2016	
				HI	MN	HI	MN	HI	MN	HI	MN
15.8SH2	62/M	Seller	32	NA	NA	**5**	**ND**	**20**	**40**	NA	NA
15.8SH46	35/M	Seller	3	NA	NA	**5**	**ND**	**20**	**40**	NA	NA
15.8SH46	66/F	Seller	8	NA	NA	**5**	**ND**	**20**	**40**	NA	NA
16.1JS24	65/M	Seller	2	NA	NA	NA	NA	**5**	**ND**	**20**	**40**

*Bold text represents titers exhibiting seroconversion. HI, hemagglutinin inhibition assay; ID, identification; MN, microneutralization assay; NA, not available; ND, not detected.

Appendix Table 8. Participants with seroconversion and persistently positive titers for avian influenza H7N9, China, 2014–2016*

Participant ID	Age, y/sex	Occupation	Length of exposure, y	Dec 2014 HI	Dec 2014 MN	Apr 2015 HI	Apr 2015 MN	Dec 2015 HI	Dec 2015 MN	Apr 2016 HI	Apr 2016 MN
Seroconversion											
14.11JS79	46/F	Feeder	10	**5**	**ND**	**40**	**20**	NA	NA	NA	NA
14.11JS77	59/F	Feeder	23	**5**	**ND**	**80**	**20**	NA	NA	NA	NA
14.11JS108	54/M	Transporter	10	**5**	**ND**	**40**	**40**	NA	NA	NA	NA
14.11GD63	23/M	Seller	5	**5**	**ND**	**20**	**20**	5	ND	NA	NA
14.12SH6	29/M	Seller	5	**5**	**ND**	NA	NA	NA	NA	**40**	**20**
15.8SH48	31/M	Seller	2	NA	NA	**5**	**ND**	**20**	**20**	NA	NA
15.8SH25	23/M	Seller	3	**5**	**ND**	**20**	**40**	NA	NA	NA	NA
16.1SD32	46/F	Processor, seller	20	NA	NA	NA	NA	**10**	**ND**	**40**	**320**
16.1JS110	27/M	Seller	13	NA	NA	NA	NA	**5**	**ND**	**20**	**80**
Persistently positive											
14.11JS73	45/M	Seller	10	20	160	20	160	NA	NA	NA	NA
14.11JS22	44/M	Seller	8	40	80	40	80	NA	NA	NA	NA
14.11JS11	29/M	Seller	10	20	40	20	80	NA	NA	NA	NA
14.11JX100	46/F	Seller	10	20	80	20	80	NA	NA	NA	NA
14.12HN48	47/F	Seller	18	20	160	20	80	NA	NA	NA	NA
14.11JX60	42/F	Seller	3	80	40	80	40	NA	NA	NA	NA
14.11JS27	34/F	Processor, seller	7	20	40	20	40	NA	NA	NA	NA
14.11JSWX25	47/F	Seller	8	20	40	40	40	NA	NA	NA	NA
14.11JSWX7	35/F	Seller	5	80	80	80	160	NA	NA	NA	NA
14.11JSWX11	47/F	Seller	10	20	80	20	80	NA	NA	NA	NA
14.11JS76	56/F	Feeder	22	80	40	40	40	NA	NA	NA	NA
14.12HN52	46/F	Seller	6	40	320	80	160	NA	NA	NA	NA
16.1JS24	46/F	Feeder	2	NA	NA	NA	NA	20	160	40	160
15.1SH23	37/F	Seller	8	20	20	NA	NA	NA	NA	40	40
14.12GD1	47/F	Seller	18	20	40	20	40	5	ND	5	ND

*Bold text represents titers exhibiting seroconversion. HI, hemagglutinin inhibition assay; ID, identification; MN, microneutralization assay; NA, not available; ND, not detected.

Appendix Table 9. Participants with seroconversion and persistently positive titers for avian influenza H9N2, China, 2014–2016*

Participant ID	Age, y/sex	Occupation	Length of exposure, y	Dec 2014 HI	Dec 2014 MN	Apr 2015 HI	Apr 2015 MN	Dec 2015 HI	Dec 2015 MN	Apr 2016 HI	Apr 2016 MN
Seroconversion											
14.11JS102	33/F	Feeder	3	**10**	**20**	**40**	**80**	NA	NA	NA	NA
14.11JS114	43/F	Transporter	16	NA	NA	NA	NA	**10**	**ND**	**40**	**160**
14.11JS8	57/M	Processor, seller	25	**10**	**40**	**40**	**80**	NA	NA	NA	NA
14.11JS95	61/M	Feeder	15	NA	NA	NA	NA	**10**	**160**	**40**	**80**
14.11JSWX45	50/F	Feeder	25	NA	NA	NA	NA	**5**	**ND**	**160**	**320**
14.12GD4	40/F	Seller	10	20	40	**10**	**ND**	**80**	**160**	**20**	**80**
14.12GD11	38/F	Seller	11	5	ND	**5**	**ND**	**40**	**160**	NA	NA
14.12GD53	49/M	Processor	11	40	40	**10**	**ND**	NA	NA	**40**	**80**
14.12GD72	34/M	Seller	5	20	80	**20**	**80**	**80**	**320**	40	160
14.12SC39	55/F	Processor	1	5	ND	**10**	**ND**	**80**	**80**	20	80
14.12SC7	42/M	Seller	5	10	ND	NA	NA	**5**	**ND**	**80**	**80**
14.12GD17	31/M	Seller	2	10	ND	**10**	**ND**	**40**	**80**	5	ND
15.5GD91	33/M	Seller	3	NA	NA	**10**	**ND**	**40**	**80**	80	640
15.8SH64	54/M	Processor	6	NA	NA	**10**	**ND**	**40**	**80**	NA	NA
16.1GD1	40/F	Seller	6	NA	NA	NA	NA	**5**	**ND**	**80**	**320**
16.1GD21	33/F	Seller	1	NA	NA	NA	NA	**5**	**ND**	**40**	**160**
16.1GD40	42/F	Seller	8	NA	NA	NA	NA	**5**	**ND**	**40**	**160**
16.1GD74	28/M	Seller	5	NA	NA	NA	NA	**5**	**ND**	**40**	**160**
16.1JS30	62/M	Feeder	1	NA	NA	NA	NA	**5**	**ND**	**40**	**80**
16.1JS51	47/M	Feeder	1	NA	NA	NA	NA	**5**	**ND**	**40**	**160**
16.1JS57	44/M	Feeder	2	NA	NA	NA	NA	**5**	**ND**	**40**	**160**
16.1SD76	39/M	Processor, seller	17	NA	NA	NA	NA	**10**	**ND**	**80**	**160**
16.5GD99	30/F	Seller	3	NA	NA	NA	NA	**5**	**ND**	**40**	**160**
Persistently positive											
14.11JS22	44/M	Seller	8	40	160	40	320	NA	NA	NA	NA
14.11JS3	53/M	Seller	20	40	160	40	160	NA	NA	NA	NA
14.11JS31	30/F	Processor, seller	3	40	640	40	320	NA	NA	NA	NA
14.11JS5	47/F	Processor, seller	15	NA	NA	NA	NA	40	160	40	640
14.11JS85	50/M	Feeder	6	40	160	40	80	NA	NA	NA	NA
14.11JS89	52/M	Feeder	9	40	160	40	80	NA	NA	NA	NA
14.11JSWX06	28/M	Seller	5	160	160	160	320	NA	NA	NA	NA
14.11JSWX7	35/F	Seller	5	40	160	80	160	NA	NA	NA	NA
14.11JSWX8	47/F	Seller	10	40	320	80	320	NA	NA	NA	NA
14.11JX100	46/F	Seller	10	40	320	40	160	NA	NA	NA	NA
14.11JX66	48/F	Seller	8	80	320	80	640	NA	NA	NA	NA

Participant ID	Age, y/sex	Occupation	Length of exposure, y	Dec 2014		Apr 2015		Dec 2015		Apr 2016	
				HI	MN	HI	MN	HI	MN	HI	MN
14.11SC28	59/F	Seller, feeder	8	40	320	80	160	NA	NA	NA	NA
14.12GD1	47/F	Seller	8	40	80	40	80	10	ND	20	160
14.12GD23	28/F	Seller	6	40	160	80	160	NA	NA	NA	NA
14.12GD24	33/F	Seller	10	80	160	80	80	5	ND	NA	NA
14.12GD3	40/F	Seller	9	80	ND	80	320	NA	NA	NA	NA
14.12HN102	34/M	Other	6	40	160	40	160	NA	NA	NA	NA
14.12HN52	46/F	Processor, seller	6	40	320	40	80	NA	NA	NA	NA
15.5GD113	35, M	Seller	0.5	NA	NA	40	80	NA	NA	40	160
15.5SC143	31/F	Seller	6	NA	NA	80	80	80	160	NA	NA
15.8SH12	50/F	Seller	30	NA	NA	80	320	40	640	NA	NA
15.8SH62	48/F	Seller, feeder	17	NA	NA	40	80	40	160	NA	NA
16.1JS101	49/M	Seller	7	NA	NA	NA	NA	80	320	80	320
16.1JS102	46/M	Seller	9	NA	NA	NA	NA	40	160	40	160
16.1JS107	42/F	Seller	15	NA	NA	NA	NA	40	160	40	160
16.1JS109	55/F	Seller	13	NA	NA	NA	NA	160	160	160	640
16.1JS110	27/M	Seller	13	NA	NA	NA	NA	40	160	40	160
16.1JS111	49/F	Seller	13	NA	NA	NA	NA	160	640	160	640
16.1JS112	24/M	Seller	13	NA	NA	NA	NA	40	160	40	160
16.1JS120	45/F	Seller	6	NA	NA	NA	NA	40	80	40	160
16.1JS15	46/F	Feeder	4	NA	NA	NA	NA	40	160	40	160
16.1JS24	65/F	Feeder	2	NA	NA	NA	NA	40	160	40	160
16.1JS96	52/F	Seller	20	NA	NA	NA	NA	80	320	80	320
16.1SC19	45/M	Seller	1	NA	NA	NA	NA	80	160	80	80
16.1SD1	30/M	Processor, seller	1	NA	NA	NA	NA	40	160	80	640
16.1SD18	35/F	Processor, seller	15	NA	NA	NA	NA	40	160	40	80
16.1SD2	39/M	Processor, seller	10	NA	NA	NA	NA	40	80	40	80
16.1SD21	46/M	Processor, seller	6	NA	NA	NA	NA	40	160	40	80
16.1SD33	48/M	Processor, seller	20	NA	NA	NA	NA	80	160	40	160
16.1SD40	37/F	Processor, seller	13	NA	NA	NA	NA	40	320	80	160
16.1SD58	30/M	Processor, seller	5	NA	NA	NA	NA	40	160	40	80

*Bold text represents titers exhibiting seroconversion. HI, hemagglutinin inhibition assay; ID, identification; MN, microneutralization assay; NA, not available; ND, not detected.

Appendix Table 10. The number of avian influenza A isolates from live poultry markets during December 2014–December 2015, China

Province	Sampling date	Sample source	No.	H5N1, no. (%)	H5N6, no. (%)	H7N9, no. (%)	H9N2, no. (%)
Guangdong	2015	Chickens	490	0	24 (4.9)	4 (0.8)	74 (15.1)
	2015	Ducks	1,326	1 (0.08)	293 (22.1)	6 (0.5)	60 (4.5)
	2015	Environment	342	0	35 (10.2)	2 (0.6)	46 (13.5)
Total			2,158	1 (0.05)	352 (16.3)	12 (0.6)	180 (8.3)
Jiangxi	2015	Chickens	1,098	0	115 (10.5)	8 (0.7)	38 (3.5)
	2015	Ducks	735	1 (0.1)	90 (12.2)	4 (0.5)	8 (1.1)
	2015	Environment	475	1 (0.2)	99 (20.8)	3 (0.6)	56 (11.8)
Total			2,308	2 (0.09)	304 (13.2)	15 (0.7)	102 (4.4)
Shanghai	2015	Chickens	481	13 (2.7)	0	19 (3.9)	69 (14.3)
	2015	Environment	12	2 (16.7)	0	1 (8.3)	2 (16.7)
Total			493	15 (3.0)	0	20 (4.1)	71 (14.4)
Jiangsu	Dec 2014–Mar 2015	Chickens	361	10 (2.8)	5 (1.4)	18 (5.0)	42 (11.6)
	Dec 2014–Jan 2015	Ducks	28	4 (14.3)	0	2 (7.1)	0
	Dec 2014–Mar 2015	Environment	87	11 (12.6)	5 (5.7)	21 (24.1)	3 (3.4)
Total			476	25 (5.3)	10 (2.1)	41 (8.6)	45 (9.5)
Sichuan	Dec 2014–Apr 2015	Chickens	190	1 (0.5)	1 (0.5)	0	0
	Dec 2014–Apr 2015	Ducks	241	2 (0.8)	17 (7.1)	1 (0.4)	6 (2.5)
	Dec 2014–Apr 2015	Environment	85	18 (21.2)	2 (2.4)	0	0
Total			516	21 (4.1)	20 (3.9)	1 (0.2)	7 (1.4)
Henan	Apr 2015	Chickens	245	0	0	0	9 (3.7)
	Apr 2015	Ducks	11	0	0	0	5 (45.5)
Total			256	0	0	0	14 (5.5)
Total			6,207	64 (1.0)	686 (11.1)	48 (0.8)	419 (6.8)

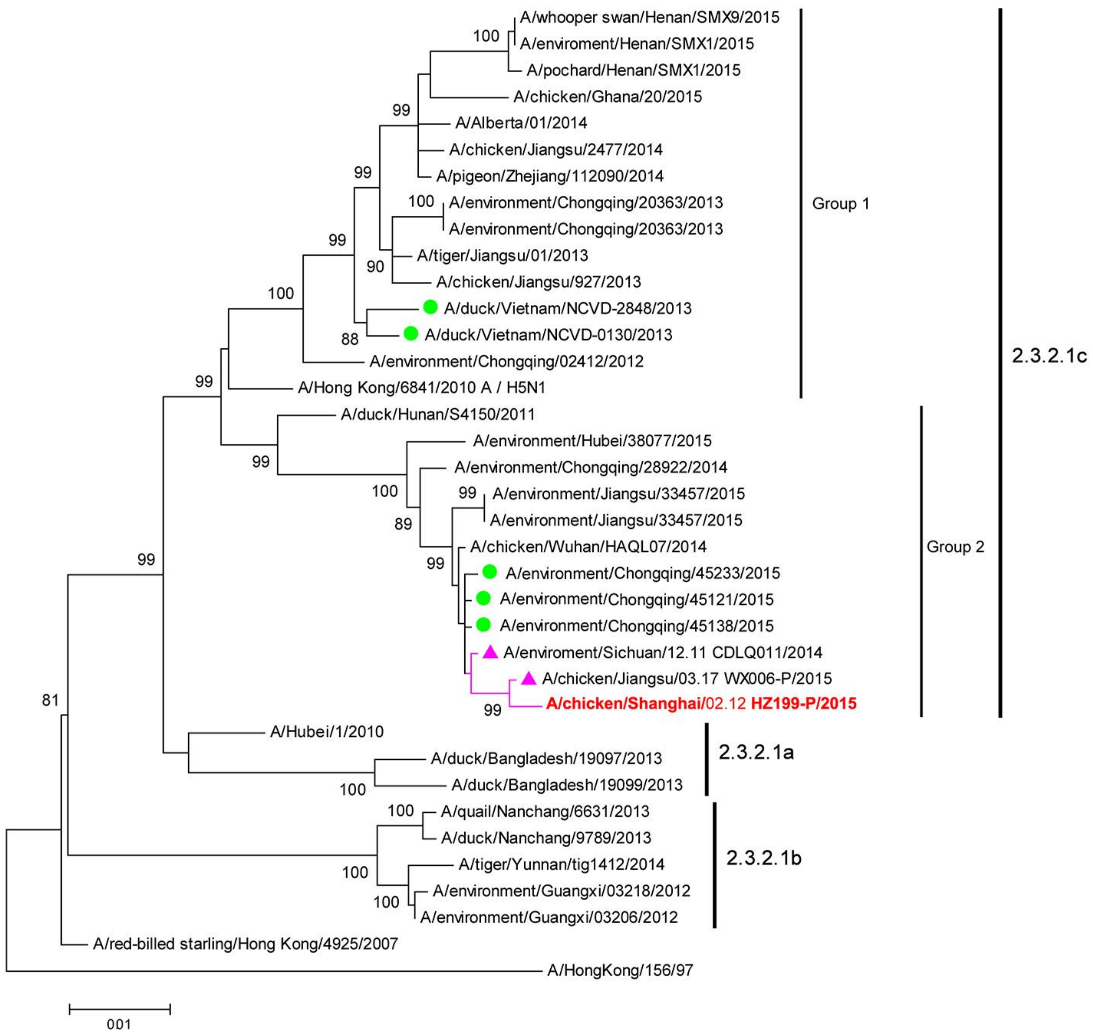

Appendix Figure 1. Phylogenetic relationship of avian influenza A(H5) clade 2.3.2.1 HA genes in a study of avian influenza viruses among occupationally-exposed poultry workers, China, 2014–2016. Green dots indicate sequences similar to recommended vaccine strains. Pink triangles indicate virus strains isolated by our laboratory. Bold text indicates human virus strains. Red text indicates reference strain used in this study. Scale bar indicates nucleotide substitutions per site.

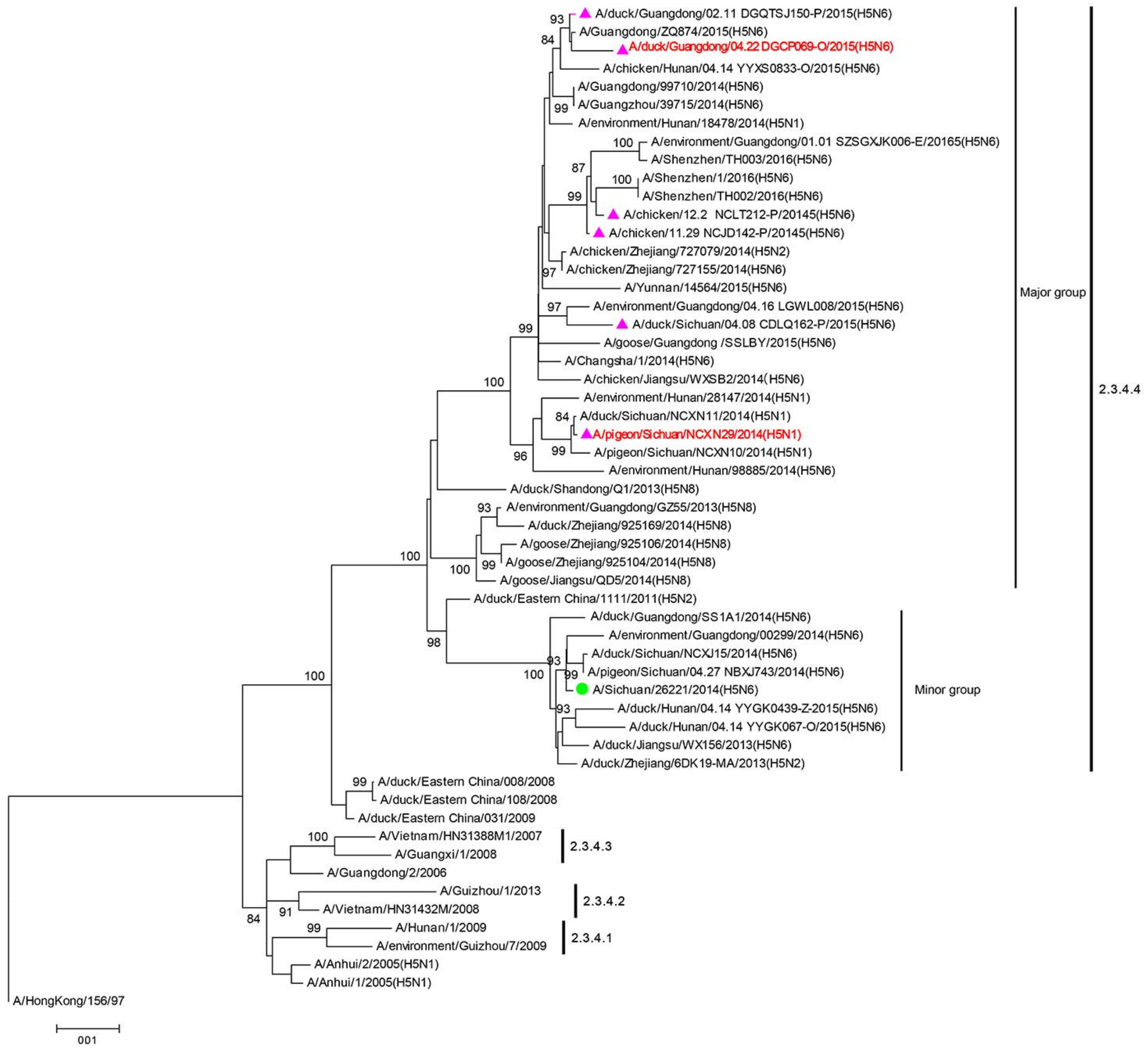

Appendix Figure 2. Phylogenetic relationship of avian influenza A(H5) clade 2.3.4 HA genes in a study of avian influenza viruses among occupationally-exposed poultry workers, China, 2014–2016. Green dots indicate available candidate influenza vaccine strains. Pink triangles indicate virus strains isolated by our laboratory. Bold text indicates human virus strains. Red text indicates reference strain used in this study. Scale bar indicates nucleotide substitutions per site.

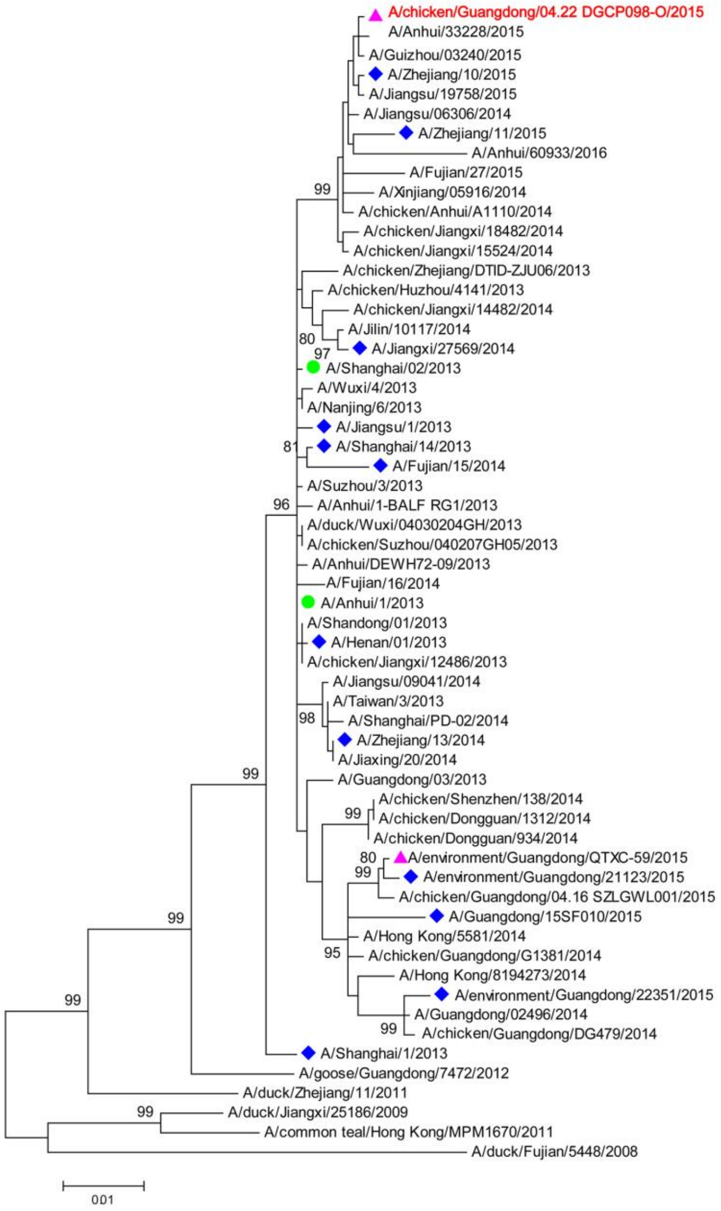

Appendix Figure 3. Phylogenetic relationship of avian influenza A(H7) HA genes in a study of avian influenza viruses among occupationally-exposed poultry workers, China, 2014–2016. Green dots indicate available candidate influenza vaccine strains. Blue diamonds indicated reported sequences from this study. Pink triangles indicate virus strains isolated by our laboratory. Bold text indicates human virus strains. Red text indicates reference strain used in this study. Scale bar indicates nucleotide substitutions per site.

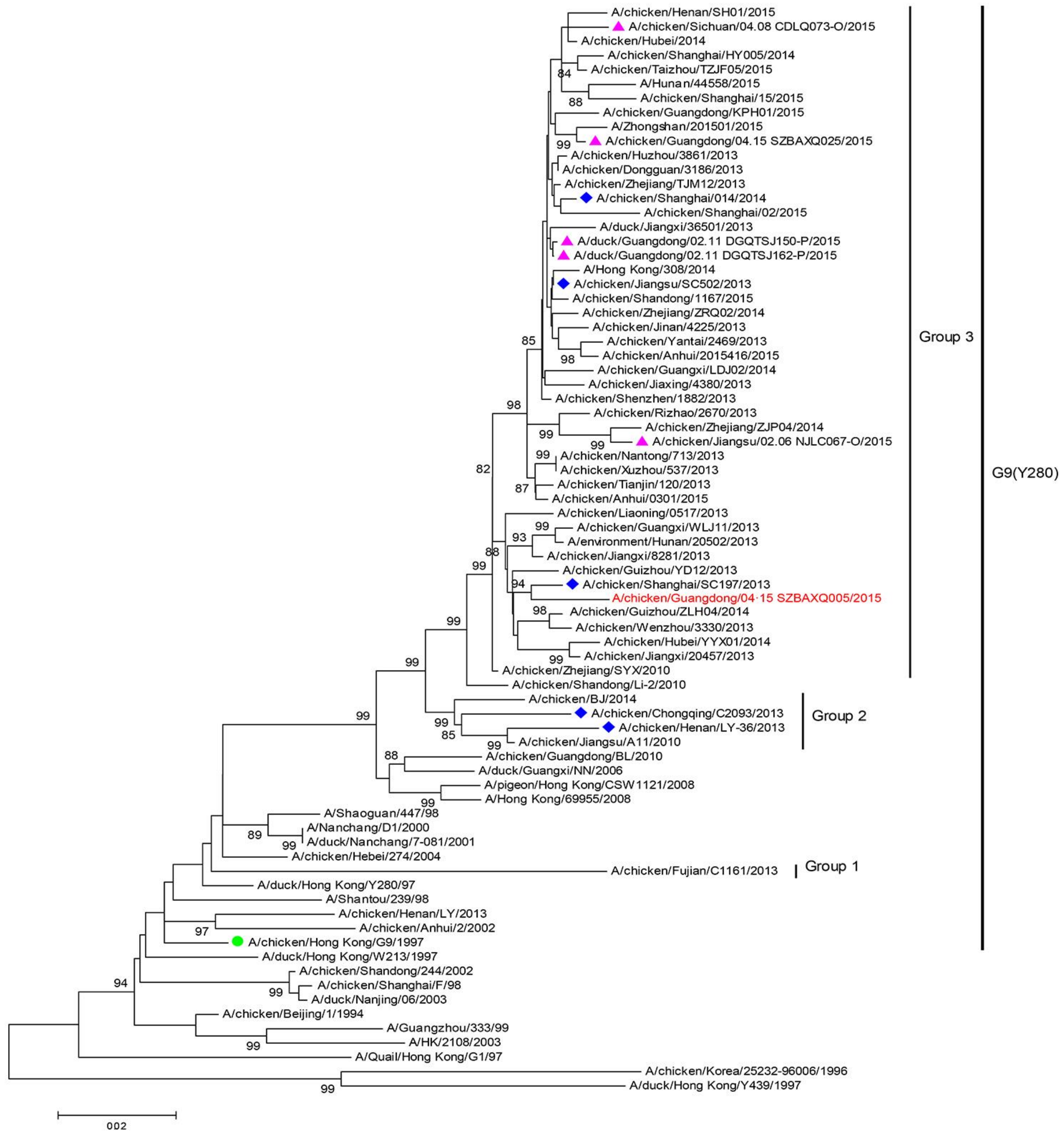

Appendix Figure 4. Phylogenetic relationship of avian influenza A(H9) HA genes in a study of avian influenza viruses among occupationally-exposed poultry workers, China, 2014–2016. Green dots indicate available candidate influenza vaccine strains. Blue diamonds indicated reported sequences from this study. Pink triangles indicate virus strains isolated by our laboratory. Bold text indicates human virus strains. Red text indicates reference strain used in this study. Scale bar indicates nucleotide substitutions per site.

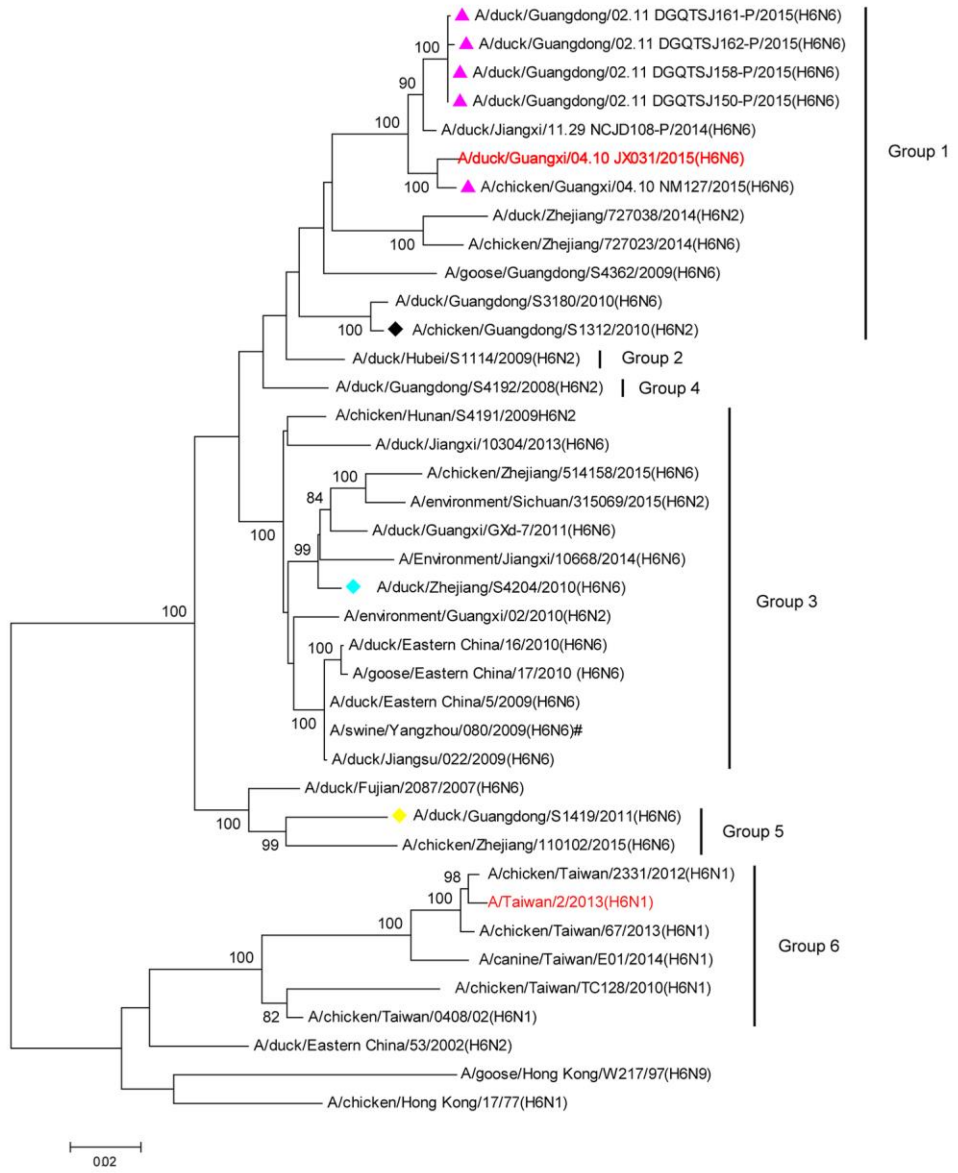

Appendix Figure 5. Phylogenetic relationship of avian influenza A(H6) HA genes in a study of avian influenza viruses among occupationally-exposed poultry workers, China, 2014–2016. Diamonds indicate antigenic characteristics reported from this study. Pink triangles indicate virus strains isolated by our laboratory. Red text indicates reference strain used in this study. Scale bar indicates nucleotide substitutions per site.
